# Mechanotransduction in the muscle spindle

**DOI:** 10.1007/s00424-014-1536-9

**Published:** 2014-06-03

**Authors:** Guy S. Bewick, Robert W. Banks

**Affiliations:** 1 School of Medical Sciences, Institute of Medical Sciences, University of Aberdeen, Aberdeen, AB25 2ZD UK; 2 School of Biological and Biomedical Sciences, University of Durham, Durham, DH1 3LE UK

**Keywords:** Muscle spindle, Mechanotransduction, DEG/ENaC, PLD-mGluR, Synaptic-like vesicle, Mechanosensation

## Abstract

The focus of this review is on the principal sensory ending of the mammalian muscle spindle, known as the primary ending. The process of mechanosensory transduction in the primary ending is examined under five headings: (i) action potential responses to defined mechanical stimuli—representing the ending's input–output properties; (ii) the receptor potential—including the currents giving rise to it; (iii) sensory-terminal deformation—measurable changes in the shape of the primary-ending terminals correlated with intrafusal sarcomere length, and what may cause them; (iv) putative stretch-sensitive channels—pharmacological and immunocytochemical clues to their identity; and (v) synaptic-like vesicles—the physiology and pharmacology of an intrinsic glutamatergic system in the primary and other mechanosensory endings, with some thoughts on the possible role of the system. Thus, the review highlights spindle stretch-evoked output is the product of multi-ionic receptor currents plus complex and sophisticated regulatory gain controls, both positive and negative in nature, as befits its status as the most complex sensory organ after the special senses.

**Electronic supplementary material:**

The online version of this article (doi:10.1007/s00424-014-1536-9) contains supplementary material, which is available to authorized users.

## Introduction

**Fig. 1:**
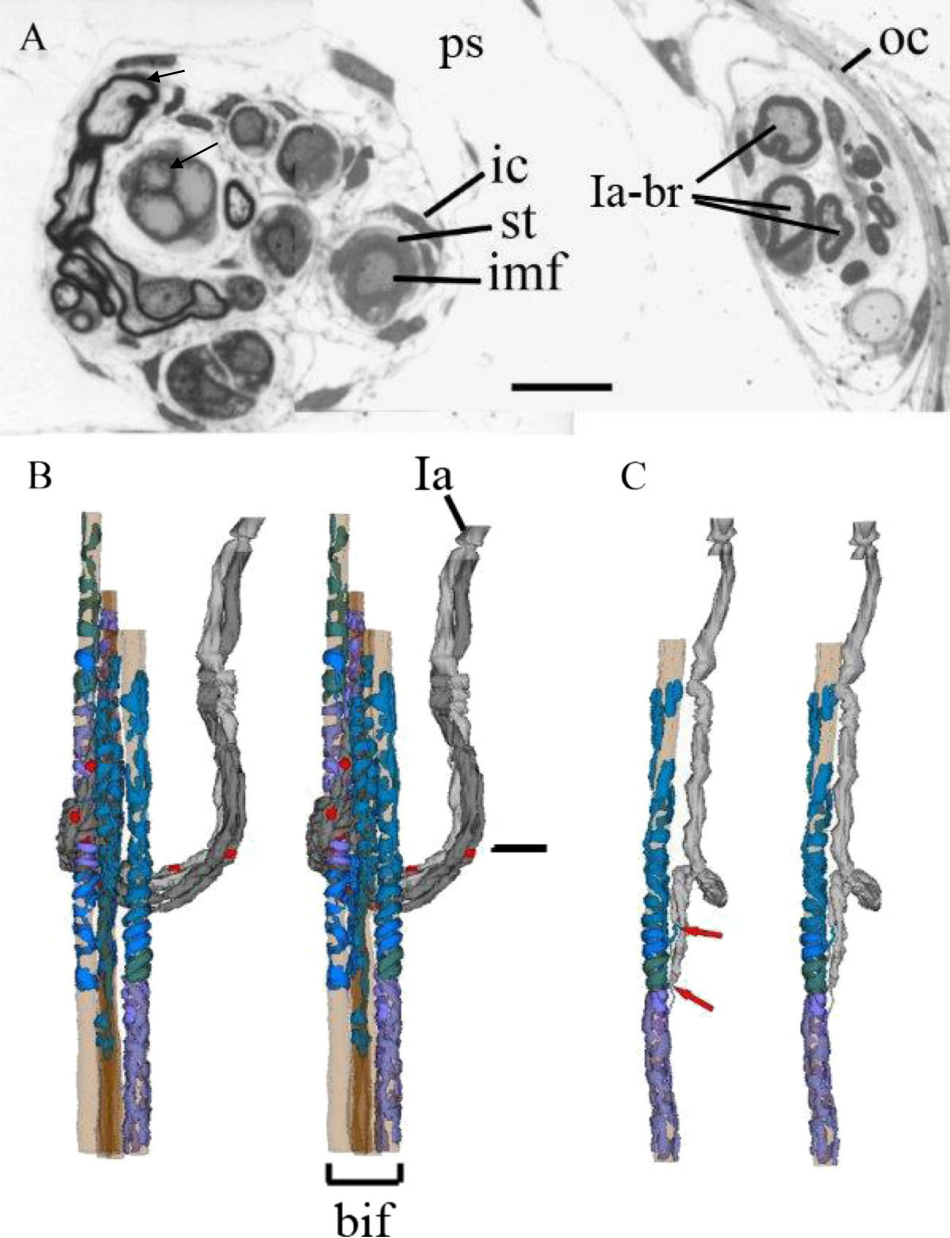
The structure of the primary ending and its enclosing capsules, as illustrated by a representative transverse section (**a**; cat tenuissimus, 1-μm-thick section, toluidine blue stain; *Ia-br* myelinated banches of the Ia parent axon, *ic* inner capsule, *imf* intrafusal muscle fibre, *oc* outer capsule, *ps* periaxial space, *st* sensory terminal, *short arrow* myelinated Ia axon, *long arrow* nuclei of intrafusal nuclear bag fibre; *scale bar* = 20 μm.) and by reconstruction (**b**, **c**) from serial transverse sections, including that in (**a**). **b** Stereopair of complete ending, with terminals in shades of blue/violet distributed, by repeated branching of the parent Ia afferent nerve fibre (*Ia* Ia parent axon with myelin in two shades of *grey*; Schwann cell nuclei in *red*), to the seven intrafusal muscle fibres present in this case (*bif* bundle of intrafusal muscle fibres). The *horizontal bar* indicates the position of the transverse EM section shown in (**a**). **c** Stereopair of one of the first-order branches of the Ia afferent, its two second-order branches each with a heminode (*arrows*) and its sensory terminals distributed to one of the intrafusal muscle fibres. Total length of reconstruction (**b**, **c**) is 365 μm

In 1926, Adrian and Zotterman [1] published one of the landmark papers in neuroscience. They showed that the response of a single-sensory end-organ to a defined stimulus was transmitted along the afferent nerve fibre in the form of a series of individual action potentials, each of fixed size, whose rate of occurrence varied according to the strength of the input stimulus. They had uncovered a general principal of the organisation of nervous systems—the way in which almost all neurons communicate over long distances, by means of a frequency code of action potentials. The end-organ that was the subject of these studies, and that therefore holds a special place in the history of neuroscience, was the frog muscle spindle, in essence a mechanosensory length transducer. Vertebrate, especially mammalian, muscle spindles are the most complex sensory organ after the special senses of the eye and the ear (for a comparative review of vertebrate muscle receptors, see [12]). A single muscle spindle receives one or more sensory nerve fibres, whose endings are located more or less in the middle of a small bundle of specialised muscle fibres (Fig. 1). These intrafusal fibres also receive their own motor innervation, allowing phasic and tonic aspects of the sensory responses to be independently adjusted (for a review of the structure and function of mammalian muscle spindles, see [9]).

Adrian and Zotterman [1] deliberately chose the muscle spindle to study, at least in part because of the relative simplicity and reliability of its stimulation by defined muscle stretches. In the decades since their fundamental observations, much has been learnt of the way in which the muscle spindle transduces changes in muscle length, brought about by stretching or shortening, though the molecular aspects of the process remain largely unknown despite some tantalising clues (again, see [9] for review). Mechanotransduction, indeed sensory transduction in general, is often treated as a simple feedforward pathway such as: stimulus → receptor potential → generator potential → action potentials. Even phenomena like adaptation can be accounted for by incorporating additional feedforward pathways. However, feedback pathways acting as gain control mechanisms between the input and output of a sensory ending are readily conceivable [47], and one of the main aspects of our recent work concerns the demonstration of the glutamatergic nature, and functional importance, of a system of small (50 nm) synaptic-like vesicles that may be part of such a mechanism [10, 16]. The vesicles have long been known to exist in most, perhaps all, mechanosensory endings but have been generally ignored.

Our focus in this review is on the principal sensory ending of the mammalian muscle spindle, known as the primary ending. We shall examine the process of mechanosensory transduction in the primary ending under five headings: (i) action-potential responses to defined mechanical stimuli—representing the ending’s input–output properties; (ii) the receptor potential—including the currents giving rise to it; (iii) sensory-terminal deformation—measurable changes in the shape of the primary-ending terminals correlated with intrafusal sarcomere length, and what may cause them; (iv) putative stretch-sensitive channels—pharmacological and immunocytochemical clues to their identity; and (v) synaptic-like vesicles—the physiology and pharmacology of an intrinsic glutamatergic system in the primary and other mechanosensory endings, with some thoughts on the possible role of the system.

## Action-potential responses to defined mechanical stimuli

**Fig. 2:**
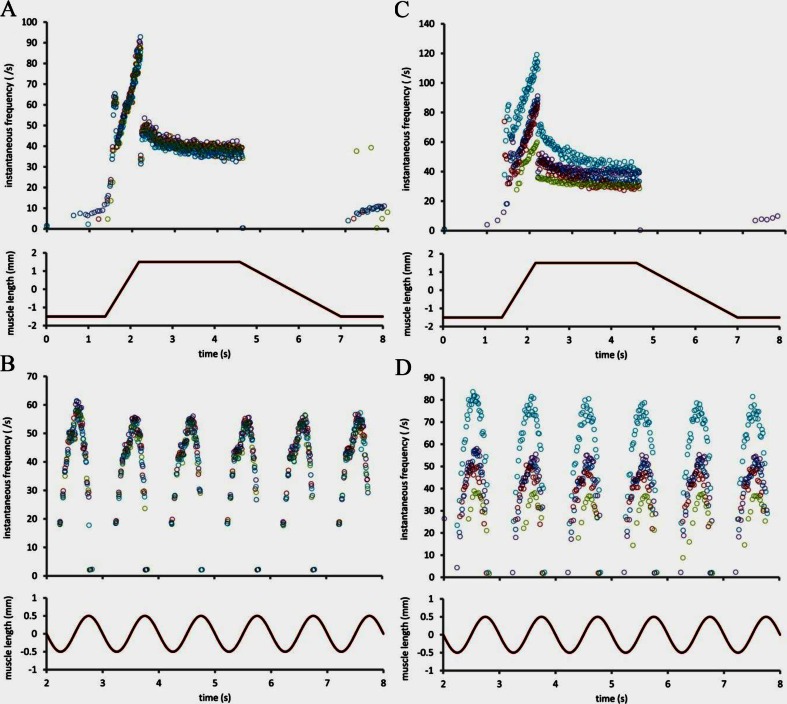
Examples of muscle-spindle primary endings responding to trapezoidal (**a**, **c**) and sinusoidal (**b**, **d**) stretches applied to the tendon of the muscle (peroneus tertius of cat). **a**, **b** The reproducibility of the responses when five separate presentations of the stimuli are given to the same primary ending. The responses are superimposed and each response is indicated by different coloured symbols. **c**, **d** The similarity of responses from five primary endings in four different preparations. The data used to construct the figure were obtained by the method given in [39] and are taken from their unpublished results. The responses are presented as plots of instantaneous frequency in which each *symbol* corresponds to a single action potential and is positioned according to the time the action potential was recorded (abscissa) and the reciprocal of the time since the previous action potential (ordinate)

This is not the place to present a comprehensive account of the responses of muscle-spindle primary endings, which would require a detailed consideration of the actions of the intrafusal motor supply as well as a wide range of mechanical stimuli defined in the time [39] or frequency domain [61]. Rather, we give just a few examples of primary endings responding to two of the most widely used patterns of stretch, trapezoidal and sinusoidal, in order to illustrate some key features of the overall transduction process (see Fig. 2). Although the responses shown here were all obtained in the absence of any concomitant motor stimulation, they are abstracted from the first few seconds of protocols that each took 40 s to complete involving much more complex patterns of combined motor and stretch stimulation [39].

We begin by noting that the responses of a single primary ending to separate presentations of the same stimulus are highly reproducible (Fig. 2a, b), provided any long-lasting mechanical after-effects arising from intrafusal motor stimulation are removed by a conditioning prestretch [11, 53]. The primary-ending response is usually considered in terms of dynamic (or phasic) and static (or tonic) components according to whether the mechanical stimulus is changing with time or not. Thus the ending is much more sensitive (here measured in impulses s^−1^ mm^−1^) to increasing length than to instantaneous length; moreover, during a decreasing length change the ending’s dynamic sensitivity must be accounted negative, allowing the output to fall to zero in some cases (Fig. 2a). Prominent features of the primary ending’s response to periodic sinusoidal stretch include phase advance and distortion (Fig. 2b), both of which may be considered to arise from the nonlinear combination of the effects of separate dynamic and static components [11].

The reproducibility not just of the pattern but of the actual firing rates of the responses of a single primary ending to separate presentations of the same stimulus may be thought remarkable enough, but when different endings, whether from separate spindles in the same muscle or from different preparations, are presented with the same stimulus the close similarity of their responses is surely even more remarkable (Fig. 2c, d). The implicit question: ‘How is the activity of the primary ending regulated so as to produce an appropriate output for a given input?’ is one to which we shall return in the sections on putative channels and synaptic-like vesicles.

## The receptor potential

**Fig. 3:**
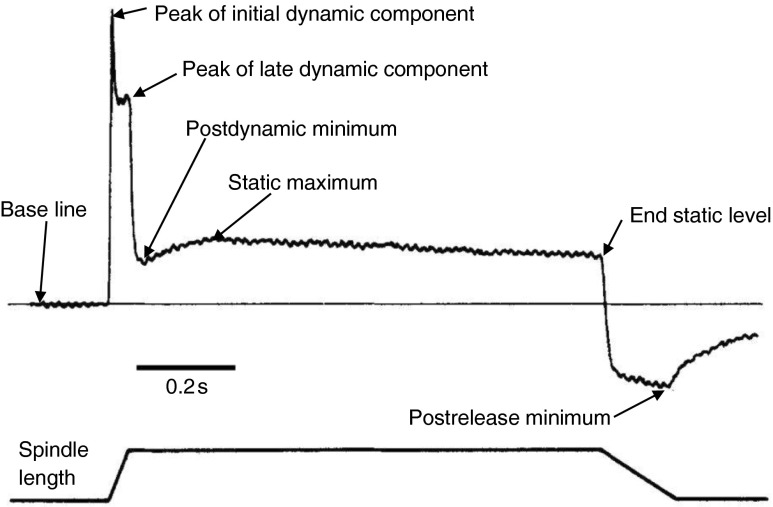
The receptor potential of a spindle primary ending (*top trace*) recorded from the Ia afferent fibre in a TTX-poisoned muscle spindle, relative depolarisation upwards, in response to a trapezoidal stretch (*lower trace*; duration of trace, 1.5 s). The various phases of the response are described according to Hunt et al. [40], who identified the pdm and the later part of the prm as due to voltage-dependent K channels [40]

**Fig. 4:**
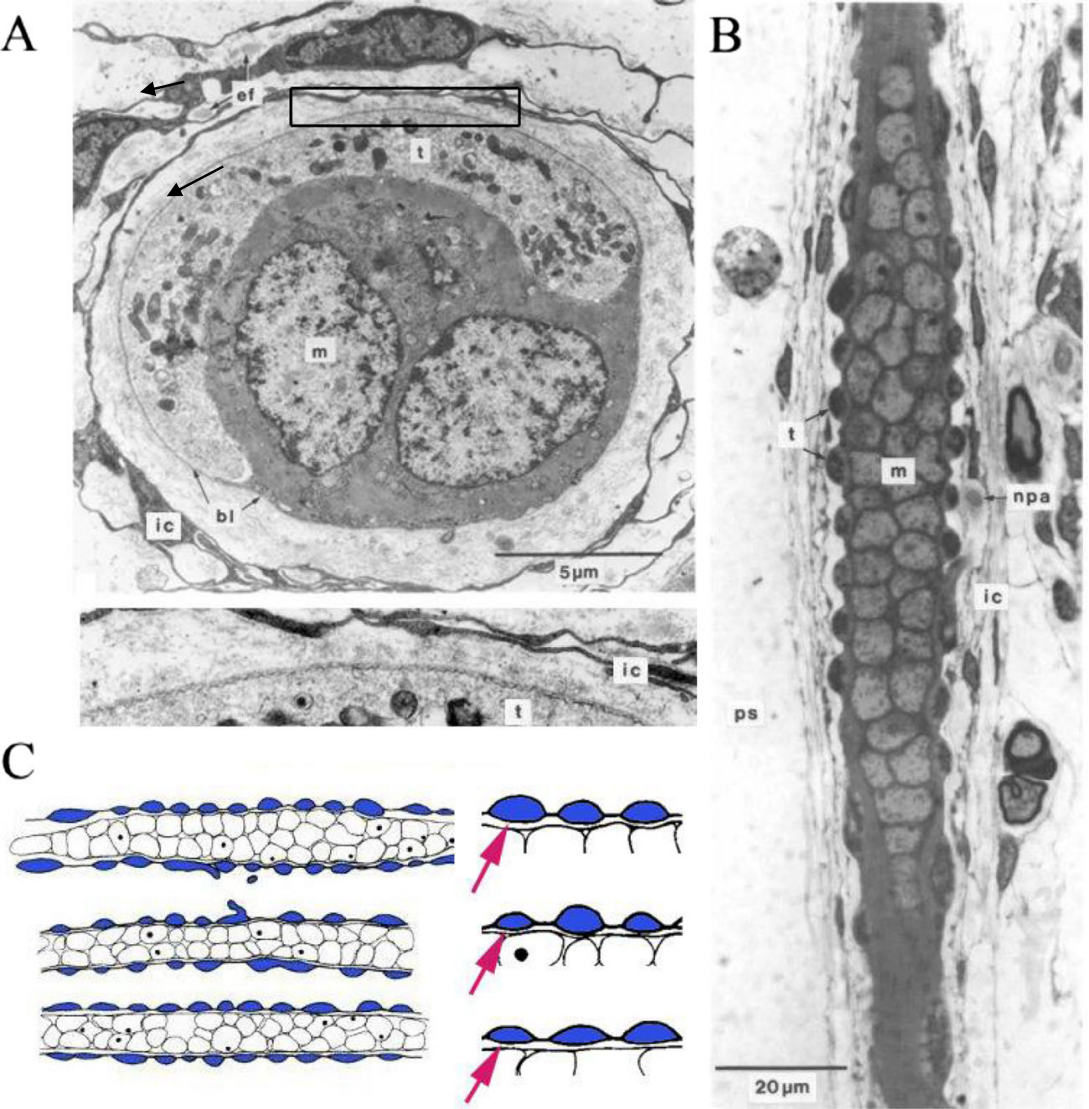
The fine structure of the sensory terminals of a spindle primary ending (**a**, **b**) and their deformation in response to maintained stretch (**c**). **a** Transverse section through an intrafusal muscle fibre (*m* label is located in one of the fibre’s myonuclei) with an enclosing sensory terminal (*t*). Note: (i) the basal lamina (*bl*) of the muscle fibre that is continuous over the outer surface of the sensory terminal and (ii) cells of the inner capsule (*ic*). Part of the sensory terminal (*black rectangle*) is enlarged below the main image to show the corrugated nature of its plasmalemma (*t*) compared with the smooth membranes of the adjacent ic cells. *ef* elastic fibres. **b** Longitudinal section through an intrafusal muscle fibre (*m* again label is located in the fibre’s myonuclei), showing the lentiform profiles of the sensory terminals (*t*) in this plane. *npa* nonmyelinated preterminal axon, *ps* periaxial space. **c** Outline tracing of the section shown in (**b**), together with similar sections through the same type of intrafusal fibre from two other spindles. Mean lengths of 50 sarcomeres on either side of the primary ending indicate that the spindles were fixed at increasing amounts of maintained tension from *top* to *bottom* (2.20-, 2.50- and 2.55-μm sarcomere lengths, respectively). Corresponding deformation of the terminal profiles was clearest in the increasing mean radii of the terminal/muscle fibre interfaces (5.2, 20.1 and 31.9 μm, respectively). Examples of representative terminal profiles are shown enlarged on the *right*, with the increased flattening of the terminal/muscle fibre interface on each fibre indicated by an arrow [8]

Direct recording of the receptor potential in the primary ending’s terminals has yet to be achieved, due mainly, perhaps, to their inaccessibility within an inner capsule (Figs. 1a and 4a, b). Equally inaccessible are the heminodes, where preterminal branches of the afferent fibre lose their myelin and where action potentials are thought to be generated (Fig. 1b, c (arrows)) [66]. Banks et al. [11] found between three and nine heminodes in each primary ending of cat tenuissimus spindles; in the more highly branched endings some of the heminodes are sufficiently distant from each other as to be effectively isolated electrotonically, allowing action potentials generated by the heminode with momentarily the highest firing rate to reset other heminodes by antidromic invasion. By eliminating action-potential firing using tetrodotoxin (TTX), and therefore allowing summation of all the receptor currents originating in the separate sensory terminals, Hunt et al. [40] succeeded in recording a continuous, stretch-dependent potential from the afferent fibre close to its exit from the spindle (Fig. 3). Depolarising receptor currents were due very largely to an influx of Na^+^, presumably through stretch-activated channels in the sensory-terminal membrane, but replacement of external Na^+^ with an impermeant cation also revealed a small, stretch-dependent, inward Ca^2+^ current. Repolarising currents probably due to K^+^ efflux were evident as receptor-potential undershoots beginning immediately after the end of a ramp stretch (postdynamic minimum (pdm)) and at the start of release of static stretch (postrelease minimum (prm)). The postdynamic undershoot appeared to be caused by voltage-gated K^+^ channels, as it could be blocked by tetraethylammonium (TEA), but the release undershoot was more complex and only a late hyperpolarisation was blocked by TEA [40]. The TEA-resistant release undershoot was not affected by removal of external Ca^2+^, or by changes in [Ca^2+^]_o_, so Hunt et al. [40] concluded that it was not caused by activation of K[Ca] channels.

In 1980, Hunt and Wilkinson [41] extended their study of mechanotransduction in the TTX-poisoned isolated muscle spindle by recording both indirect receptor potential (i.e. propagated to the axon by electrotonic spread) and tension in response to sinusoidal stretch varying in both displacement and frequency. Their results were broadly in line with those obtained some time earlier by Matthews and Stein [51] who had recorded action potentials from in situ spindles, but in addition they [41] were able to show that many of the nonlinearities such as gain compression originally described in the in situ preparation are present in both the receptor-potential and tension responses. The parallelism between the receptor potential and intrafusal tension suggests that many features of the sensory response have their source in the mechanical transmission of the stretch stimulus to the sensory terminals; however, Kruse and Poppele [47] provided compelling evidence that within the linear displacement range the midfrequency dynamics (0.4–4 Hz) did not arise from the mechanical properties of the contractile apparatus of the intrafusal muscle fibres, but rather were intrinsic properties of the sensory terminals. They explicitly identified K[Ca] channels as in part responsible for the mid-frequency dynamics by providing a negative feedback loop within the overall mechanotransduction process and in support of this, we have recently found immunoreactivity for SK2-type K[Ca] channels in the sensory terminals of muscle spindles and lanceolate endings of hair follicles (Shenton et al., unpublished data).

## Sensory-terminal deformation

Direct observation of isolated or semi-isolated muscle spindles shows that stretch of the spindle is accompanied by extension of the sensory region and measurable increase in the spacing between the turns of the primary-ending terminals [17, 62]. The sensory terminals appear to adhere to the surface of the intrafusal muscle fibres and they do not directly contact any other cellular structure. Intrafusal muscle fibres, in common with skeletal muscle fibres generally, possess an extracellular, collagenous basal lamina, which is in close contact with the plasmalemma of the muscle fibre everywhere except at the sensory terminals (Fig. 4a). Attachment of the basal lamina to the plasmalemma probably involves the dystrophin complex, and dystrophin is missing precisely where the sensory terminals intervene between the basal lamina and muscle fibre plasmalemma [54]. The basal lamina may therefore be an important structural component, helping to locate and attach the sensory terminals to the intrafusal muscle fibres. Stretch of the sensory region is accompanied by deformation of the terminals, first described in frog spindles [14]. In mammalian spindles, the profiles of sensory terminals, when cut in longitudinal section through the sensory region, present a characteristic lentiform shape that varies in relation to intrafusal-fibre type and amount of static tension (as indicated by sarcomere length, Fig. 4b, c). Analysis of the profile shapes shows that the terminals are compressed between the plasmalemmal surface of the intrafusal muscle fibres and the overlying basal lamina [8]. Assuming that the terminals are constant volume elements, this compression leads to deformation of the terminals from a condition of minimum energy (circular profile) and therefore to an increase in terminal surface area. The tensile energy transfer from the stretch of the sensory region to the terminal surface area may be proposed to gate the presumed stretch-activated channels in the terminal membrane. Well-fixed material shows a fine, regular corrugation of the lipid bilayer of the sensory terminal membrane (Fig. 4a), so it seems likely that the tensile-bearing element consists in cytoskeletal, rather than lipid bilayer, components of the membrane [8].

## Putative stretch-sensitive channels

The stretch-sensitive channel(s) responsible for transducing mechanical stimuli in spindle afferents, as in most mammalian mechanosensory endings, awaits definitive identification. Candidate mechanotrasnducer channels have been reviewed in detail recently [22]. In spindle primary terminals at least, multiple ion channel types must be responsible for generating and regulating the frequency of afferent action potentials. Hunt et al. [40] showed that in mammals while Na^+^ is responsible for ~80 % of the generated receptor potential, there is also a clear involvement of a stretch-activated Ca^2+^ current. Conversely, the postdynamic undershoot is driven by K^+^, particularly a voltage-gated K^+^ current. Finally, other studies [47, 70, 79] indicate a role for K[Ca] currents. Most, perhaps each, of these must involve opening specific channels.

**Fig. 5:**
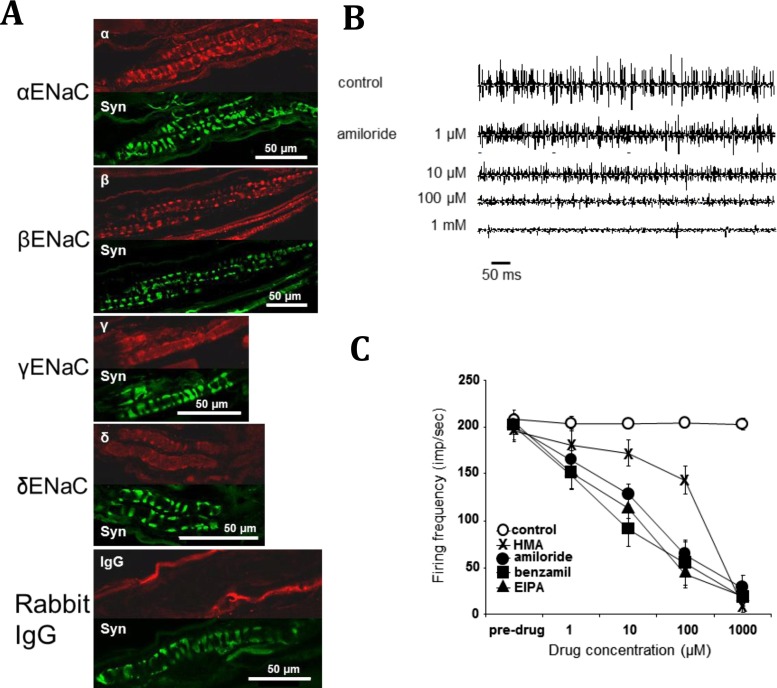
Evidence for amiloride-sensitive ENaC family members in spindle sensory terminals. **a** Confocal immunofluorescence images of labelling for α, β, γ and δ ENaC (*red*) localises to the sensory terminals, double-labelled with synaptophysin (*green*). Synaptophysin labels the synaptic-like vesicles in the primary sensory terminals. **b** Stretch-evoked firing is inhibited by amiloride in a dose-dependent manner, in the range of 1–1,000 μM. **c** Similar effects are seen with other amiloride analogues, except hexamethyleneamiloride (*HMA*) [71]

We will first examine the evidence surrounding the putative mechansensory channel(s) carrying Na^+^ and Ca^2+^ currents. It seems unlikely the whole receptor current is supported by a single type of nonselective cation channel, as Ca^2+^ is unable to substitute for Na^+^ in the receptor potential [40]. Members of three major channel families have been proposed as the mechanosensory channel; degenerin/epithelial Na channels (DEG/ENaC), transient receptor potential (TRP) superfamilies [56, 74] and piezos [20]. There is strong evidence for TRP channels as neural mechanosensors in invertebrates, particularly *Drosophila* [33, 56, 74]. However, there is little evidence for a role in low-threshold sensation in spindles. Strong evidence against them being the major driver of spindle receptor potential is they either display high Ca^2+^ selectivity or pass Na^+^ and Ca^2+^ equally well. While piezos 1 and 2 certainly contribute to mechanical responses to nociceptive touch in mammalian sensory neurones, they are nonselective cation channels and there is again no strong evidence for their presence in spindles [20]. Finally, however, there is mounting evidence in mammalian primary afferent neurones, and in the sensory endings of spindles in particular, for the involvement of members of the DEG/ENaC superfamily as mechanosensory channel(s) [4, 44, 67, 68, 71]. Importantly, many channels in this family are highly selective for Na^+^ over Ca^2+^ and K^+^ [32]. However, their role as stretch-activated channels is disputed [67]. Attempts to show mechanical activation in heterologous systems have been unsuccessful [7, 67], but this may reflect a block by intracellular ATP [49]. We have produced evidence for all four subunits of the ENaC channel (α, β, γ and δ) in spindle primary-sensory terminals, by pharmacology, immunofluorescence and Western blotting (Fig. 5) [71]. ENaC channels are thought to be heterotrimers [45], of either α, β and γ or δ, β and γ composition, with the α or δ subunits forming the pore. Another superfamily member are the acid sensitive ion channels (ASICs), where ASIC1a/b, 2a/b, 3 or 4 make up the pore, probably in homo/heterotrimeric combination with each other or even ENaC β and γ [45]. Their role in wider sensory perception has been extensively reviewed elsewhere [48]. Spindle sensory terminals were indeed immunofluorescent for ASIC2a. All ENaC/ASIC labelling in spindle mechanosensory terminals strongly colocalised with synaptophysin, a marker for the synaptic-like vesicles (SLVs) regulating afferent excitability (see next section). Thus, the channels may be stored in intracellular vesicular compartments and delivered to the terminal membrane by vesicle fusion. This would be consistent with inhibition by syntaxin 1A of ENaC currents when these proteins are co-expressed in *Xenopus* oocytes [64] and with vesicle-associated localisation of immunogold ENaC labelling in rat kidney epithelium, where ENaCs regulate Na fluxes [36].

All ENaCs/ASICs are inhibited by amiloride, and we found spindle stretch-evoked outputs are highly amiloride sensitive. However, there was little discrimination between amiloride analogues, with the exception of hexamethyleneamiloride, which had a particularly steep dose/response relationship (Fig. 5b, c). More recently, we have explored the potential of other ENaCs as mechanotransducers. When expression was linked to green fluorescent protein, ASIC3-associated fluorescence was found in spindle primary sensory terminals. Our functional studies used a novel assay of stretch-evoked responses in neurites from ASIC3 expressing large-diameter dorsal root ganglion neurones (DRGs). Deformation of the flexible substrate underlying the neurites, to simulate low-threshold activation, reliably evoked action potentials in an amiloride-sensitive manner. Moreover, DRG-specific ASIC3 deletion ablated this substrate indentation-evoked response. Interestingly, neither amiloride nor gene deletion affected responses to direct contact-stimulated responses, perhaps analogous to nociceptive stimulation. Finally, DRG-targeted ASIC3 knock out produced significant deficits in fine proprioceptive tasks in vivo, analogous to the light-touch deficits in ASIC2a knockouts [63]. Overall, these findings implicate low affinity ENaCs as a major component of the spindle mechanotransducer. The presence of multiple channel isoforms, together with the absence of dramatic effects following knock out of single genes, suggests that either yet another whole class of channels remains to be discovered, or there is great functional redundancy in the channels expressed or, perhaps most likely, the primary mechanotransducer channels are composed of heteromultimers from various member of the ENaC superfamily, analogous to the MEC channels in *Caenorhabditis elegans* [5].

The next question is which channel(s) support the Ca^2+^ current. While Ca^2+^ only contributes ~20 % of the receptor current, it is clearly functionally essential. Removal of extracellular Ca^2+^ or application of the inorganic voltage-gated channel blocker Co^2+^ rapidly block stretch-evoked firing [16, 47]. We have found similar effects with Ni^2+^/Cd^2+^. As ENaC (α, β and γ) channels have a very high Na^+^ selectivity, passing almost no Ca^2+^, another channel must be responsible for the substantive Ca^2+^ component to the receptor potential [40]. Several candidates have been suggested, including ASICs, TRPs and even voltage-gated Ca^2+^ channels. The Ca^2+^ selectivity or cation nonselectivity of TRP channels makes them candidates, but there has been no systematic study of TRP channels in spindles. If present, it is unlikely to be TRPV1 and TRPM8, as we find the TRPV1 antagonist capsazepine [13] actually enhances stretch-evoked firing in spindles. Conversely, icilin, a particularly potent TRPM8 agonist [13, 77], increases firing only modestly [71]. Other candidate TRP channels include members of the TRPC family, where a number of reports suggest they are associated with mechanotransduction in other cell types, e.g. [30, 35, 69, 72, 73]. However, expression in heterologous systems does not support a role for them directly in mechanotransduction [35] but rather in Ca^2+^ release from intracellular compartments [33]. Of the ASICs, only ASIC1a is known to be significantly permeable to Ca^2+^, and its presence in spindle endings has not been reported. Thus, while a Ca^2+^-permeable, stretch-activated channel is clearly present, its identity is unclear.

There is, however, significant evidence of important functional roles for voltage-gated Ca^2+^ and K[Ca] channels in modulating stretch-evoked spindle output [47]. L-type voltage-activated Ca^2+^ channels may indeed contribute to the receptor potential and/or the encoding process, as high nifedipine concentrations inhibit firing [29]. N-type channels have been reported to exhibit mechanical sensitivity in heterologous systems [18]; however, we found the N-type channel toxin ω-conotoxin GVIA had no effect on firing [70].

Interestingly, antagonists of the remaining Ca^2+^ channels tested, and the K[Ca] channels, all increase firing. Thus, Zn^2+^ (T-type channel blocker) [47] and ω-agatoxin IVA (P/Q-type) [70] both enhanced spindle firing. In fact, P/Q channel blockade increased firing rates quite profoundly, to some 300 % of basal rates. This indicates that rather than contribute to the receptor potential, particularly P/Q-type and perhaps T-type channels help regulate firing rates. Incidentally, Zn^2+^ is also an activator of ENaC and piezo channels [34]. Thus, the increased firing may be the first evidence for piezo in spindle sensory terminals.

It seems the Ca^2+^-channel mediated regulation of firing rates is linked to activation of K[Ca] channels. K^+^ outflow by Ca^2+^-dependent opening of these channels will produce hyperpolarisation, tending to dampen firing rates below that expected directly from the depolarising receptor potential. Blocking the channels with apamin (SK), iberiotoxin, charybdotoxin, paxilline (BK) and TRAM 34 (IK), all increase firing [47, 70]. Conversely, activating the BK channel with NS1419, blocks spindle firing entirely. A complete description of this study is in preparation.

In summary, the mechanosensory channels producing the spindle receptor potential still await definitive identification. The major (~80 %) current from the mechanosensory channels is due to Na^+^. There is a minor (~20 %) contribution from Ca^2+^, also in a mechanically sensitive manner. Prime candidates responsible for the Na^+^ current are ENaCs and/or ASICs. The Ca^2+^component seems likely to flow through ASIC1a and/or L-type voltage-gated channels, although it may also involve TRP channels. Our results with SK2 suggest a direct contribution of this channel to the receptor potential (Shenton et al., unpublished data), but the remaining Ca^2+^and K[Ca] channels seem rather to be concerned with regulating the firing frequency in response to the receptor potential through T- and particularly P/Q-type channels, linked to a family of K[Ca] channels. While there is evidence for SK, IK and BK, the BK channels certainly play a major role, as their direct activation alone can entirely abolish spindle output. This relationship between P/Q-type and BK channels is reminiscent of the regulation of firing in a number of places in the nervous system. Simultaneous expression of voltage-gated Ca^2+^and K[Ca] channels to regulate neuronal excitability is common in the CNS [15, 27, 50, 80] and has also been found to control firing in a range of other peripheral mechanosensitive cell types [38, 60].

## Synaptic-like vesicles

**Fig. 6:**
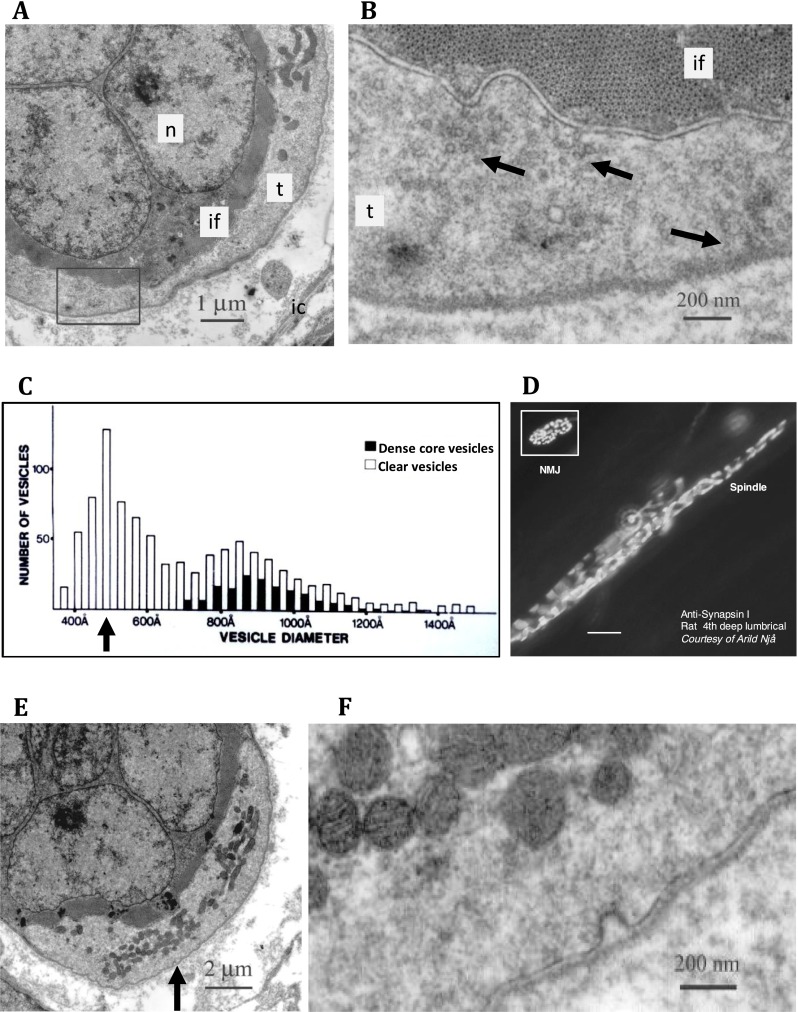
Fifty-nanometre, clear synaptic-like vesicle (*SLV*) clusters in spindle sensory terminals. **a** Electronmicrograph of a transverse section of the central portion of a nuclear bag intrafusal fibre (*if*) with its distinctive collection of prominent nuclei (*n*) and an enclosing sensory terminal (*t*). The *boxed region* is shown at higher magnification in (**b**), where distinctive clusters of synaptic-like vesicles can be seen (*arrows*), some aggregated towards and some away from, the muscle fibre. Quantification of vesicle diameters (**c**) shows the most abundant are clear and 50 nm (500 Å) in size, similar to their synaptic counterparts. Synapsin I labelling (**d**), a presynaptic vesicle-clustering protein, is present in the typical annulospiral ending of a rat lumbrical primary sensory terminal. Labelling in a motor nerve terminal in the same muscle is of similar intensity (*inset*, for comparison; *NMJ*, neuromuscular junction). Spindle terminals do not stain for synapsin II or III (Arild Njå, personal communication). *Scale bar*, 20 μm. **e**, **f** A coated pit of approximately 50-nm diameter in the axolemma of a sensory terminal, typical of endocytosis, as evidence of active SLV recycling. Note this pit is on the surface directed away from the nuclear bag fibre it encloses, although we have seen retrieval areas on both surfaces

Populations of vesicles are a prominent feature of muscle spindle primary afferent terminals at the EM level (Fig. 6a, b), as they are in all mechanosensory endings [3, 19, 83]. While these vesicles can vary in size and morphology, most are described as small and clear. When carefully quantified in spindles, the most abundant vesicle population is one of 50 nm diameter (Fig. 6c). Since the discovery of these vesicles in sensory endings, contemporaneous with their synaptic counterparts [19, 46], sporadic reports show spindle terminals also express functionally important presynaptic proteins: the vesicle clustering protein synapsin I and the ubiquitous synaptic vesicle protein synaptophysin [21] (Figs. 5a and 6d); the vesicle docking SNARE complex protein, syntaxin 1B [2]; as well as many presynaptic Ca^2+^-binding proteins (calbindin-D28k, calretinin, neurocalcin, NAP-22 and frequenin) [25, 26, 28, 37, 42, 43, 78]. Several functional similarities have emerged too, including evidence of endocytosis (Fig. 6e, f), and their depletion by black widow spider venom [64]. Despite these commonalities, the role of the vesicles was largely ignored for over 40 years, presumably due to lack of an obvious function in sensory terminals.

**Fig. 7:**
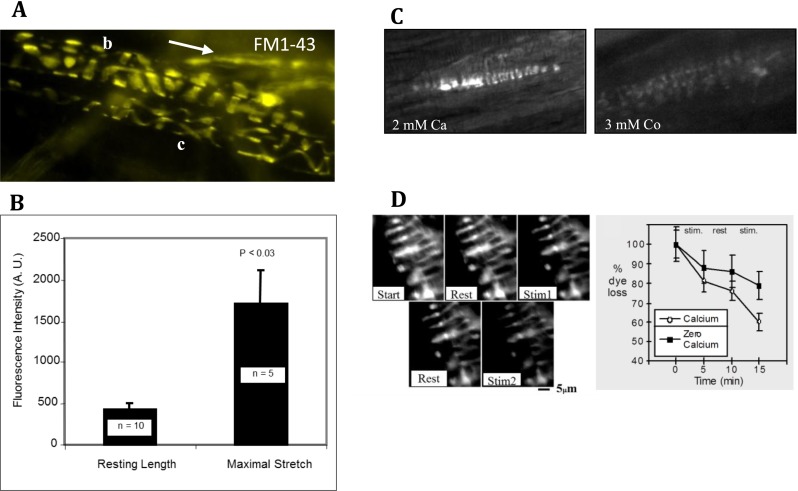
FM1-43 labelling of differentiated primary spindle endings involves local synaptic-like vesicle recycling. Spontaneous FM1-43 labelling of primary endings in adult rat lumbrical muscle (**a**), showing characteristic differences in pitch, intrafusal fibre diameter and terminal ribbon width associated with nuclear bag (*b*) and chain (*c*) fibres. Incoming IA afferent axons also sequester dye (*arrow*) independent of activity due to their high myelin content. Intrafusal fibres enclosed by the endings are translucent, as they do not take up the dye. Terminal labelling is spontaneous but greatly increased by mechanical activity (repeated maximum stretch, **b**). It is also Ca^2+^ dependent, as it is essentially eliminated by the channel blocker Co^2+^ (**c**). **d** Unlike labelling by mechanosensory channel permeation, FM1-43 labelling in differentiated spindle terminals is reversible (**d**), showing clearly enhanced destaining with vibration (*left images*, 200 Hz, 50-μm amplitude, 5 min), a process which is also Ca^2+^ sensitive (*right graph*). This is consistent with FM1-43 uptake/release in differentiated terminals through local Ca^2+^-dependent recycling of SLVs in these endings ([16], b–d)

Through uptake and release of the fluorescent dye FM1-43, we showed the vesicles undergo constitutive turnover at rest, and that turnover increases with mechanical activity (Fig. 7a, b) [16]. Unlike the stereocilia of cochlear hair cells [31], or many DRG neurones in culture [24], this labelling does not seem to greatly involve dye penetration of mechanosensory channels, as it is reversible, resistant to high Ca^2+^ solutions, and dye has little effect on stretch-evoked firing in spindles [16, 75] or indeed in other fully differentiated mechanosensory terminals [10]. Dye turnover is, however, Ca^2+^ dependent, as both uptake and release are inhibited by low Ca^2+^ and the Ca^2+^-channel blocker, Co^2+^ (Fig. 7c, d). Thus, vesicle recycling in mechanosensory terminals, as with synaptic vesicles, is Ca^2+^ dependent, constitutive at rest (*cf* spontaneous synaptic vesicle release at synapses) and is increased by activity (mechanical/electrical activity, respectively). However, these terminals are not synaptic, as vesicle clusters (Fig. 6b) and recycling (Fig. 6e, f) are not specifically focussed towards the underlying intrafusal fibres nor, apparently, around specialised release sites (RWB, unpublished data). While trophic factors are undoubtedly secreted from primary terminals to influence intrafusal fibre differentiation, these almost certainly involve larger, dense core vesicles. By contrast, turnover of the small clear vesicles is primarily modulated by mechanical stimuli applied to the terminal, making them concerned with information transfer in the opposite direction to that normally seen at a synapse.

The first strong evidence for a functional importance of vesicle recycling was the observation that stretch-evoked firing fails following tetanus toxin injection and at the same rate as neuromuscular synaptic transmission [52]. This shows the toxin’s target, synaptobrevin, essential for docking and exocytosis of synaptic vesicles, is also crucial for maintaining spindle sensitivity to stretch.

These synaptic similarities and dissimilarities led us to term the organelles ‘synaptic-like vesicles’ or SLVs. As a further similarity, we found that spindle sensory terminals contain synaptic levels of the classical neurotransmitter glutamate, while others have shown they express vesicular glutamate transporters [82] (specifically vGluT1, although not vGluT2 or vGluT3), essential for loading vesicles with glutamate neurotransmitter.

**Fig. 8:**
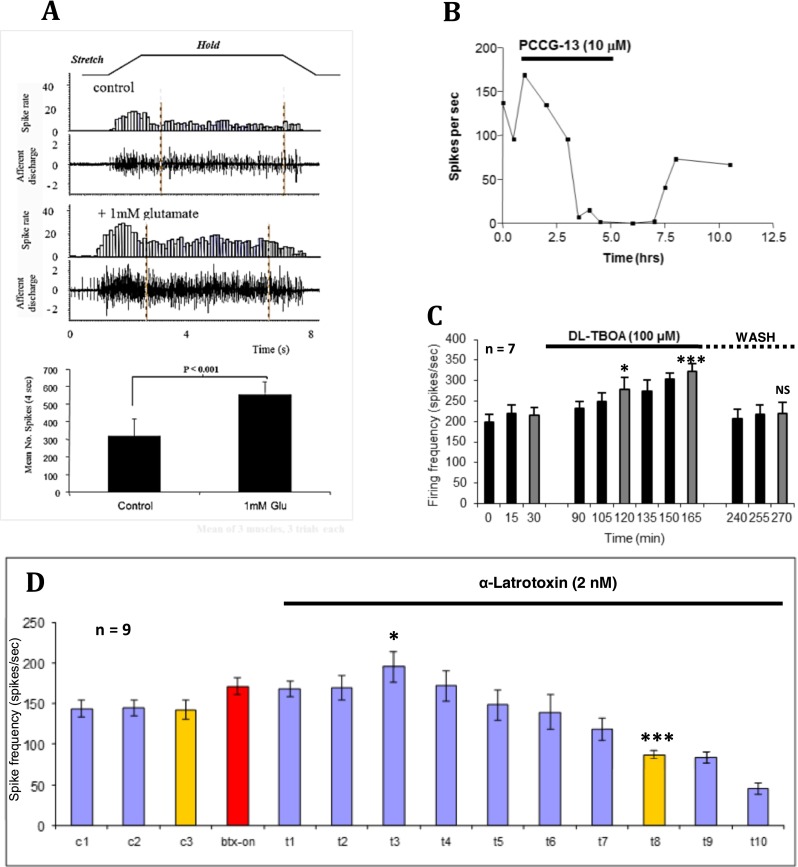
Endogenous glutamate secretion from SLVs maintains spindle stretch-evoked responsiveness. **a** A standard trapezoidal stretch-and-hold (~10 % muscle length) applied to a rat lumbrical muscle containing 8–12 muscle spindles evokes robust spiking activity in the electroneurogram (afferent discharge) and quantified in the firing frequency histogram (spike rate). Exogenous glutamate (1 h, 1 mM) can essentially double firing rate for the stretch. The histogram shows total firing within the 4-s plateau (hold phase) sample period indicated. Conversely, **b** inhibition of the highly atypical glutamate receptor with PCCG-13, applied in the absence of glutamate, can totally and reversibly block stretch-evoked spindle output. Note the timescale of hours, showing the long timecourse over which this modulation occurs. **c** Endogenous glutamate secretion occurs and is important for regulating firing, as blocking glutamate re-uptake by terminal excitatory amino acid transporters (*TBOA*), again in the absence of exogenous glutamate, enhances firing just as effectively as application of exogenous glutamate. *P < 0.05; ***P < 0.0001 vs. 30-min control firing (*grey bars*). 1- to 2-h wash reverses this effect (*NS*, not significantly different from pre-TBOA control). **d** Endogenous glutamate secretion is from SLVs. α-Latrotoxin, which evokes uncontrolled vesicle release, and ultimately vesicle depletion from spindle and synaptic endings [64], initially enhances stretch-evoked firing (*P < 0.05) then inhibits firing (***P < 0.0001), as SLVs are first released, then depleted. *c1–c3* are recorded every 15 min, while *t1–t10* are recorded at 30-min intervals. *Btx-on* bungarotoxin was first applied for 30 min prior to α-latrotoxin, to block spontaneous mechanical stimulation by fibre contraction driven by the α-latrotoxin-stimulated ACh secretion from fusimotor and extrafusal synaptic motor nerve terminals ([16], a, b)

Subsequently, we found SLVs are part of an activity-regulated glutamate secretory system that is required to maintain normal spindle responses. Exogenous glutamate can double the stretch-evoked firing rate (Fig. 8a), while glutamate receptor antagonists can both inhibit this glutamate-mediated increase and, importantly, reduce firing if applied alone (Fig. 8b). Indeed, prolonged exposure (>4 h) can entirely, and reversibly, abolish stretch-evoked firing altogether. The latter observation and the blockade by tetanus toxin are strong evidence that glutamate release from SLVs is essential for maintaining spindle responsiveness, and over a prolonged timescale of hours, rather than directly involved in mechanotransduction itself. More recently, we have strengthened the evidence for endogenous glutamate secretion by showing that simply blocking membrane glutamate transporters with dl-threo-β-benzyloxyaspartic acid (TBOA; Fig. 8c) markedly increases stretch-evoked firing, presumably through extracellular accumulation of secreted glutamate. This endogenous secretion seems to involve SLVs, as responsiveness is inhibited by blocking exocytosis with Ca^2+^ channel inhibitors (Co^2+^ and Ni^2+^/Cd^2+^, see earlier). Moreover, stimulating exocytosis to deplete SLVs with α-latrotoxin [65] initially enhances, then inhibits firing (Fig. 8d). This action of α-latrotoxin, the active ingredient of black widow spider venom, highlights another presynaptic similarity of spindle afferent terminals; they must express the latrotoxin receptor latrophilin and/or neurexin.

Another intriguing aspect of spindle glutamate sensitivity is the highly unusual pharmacology of the receptor involved. It is resistant to classical ionotropic (iGluR; kynurenate) or metabotropic (mGluR) receptor antagonists (MCPG (groups I and II) or CPPG and MAP4 (group III)), even applied together [16]. However, it is actually inhibited by the classical group I agonist R,S-DHPG, and particularly by PCCG-13 (Fig. 8b). PCCG-13 was developed by Pellicciari and colleagues [59] as a selective antagonist of a novel mGluR in the hippocampus, coupled to phospholipase D activation (PLD) [6, 58]. We have confirmed the highly atypical and non-canonical pharmacology of the spindle mGluR in a recently completed extensive pharmacological investigation [76]. The mGluR-mediated increase in responsiveness in spindles is also linked to PLD, as the PLD inhibitor FIPI blocks both the response to exogenous glutamate and inhibits spindle firing when applied alone, in a dose-dependent manner. We have therefore termed this receptor the PLD-mGluR.

**Fig. 9:**
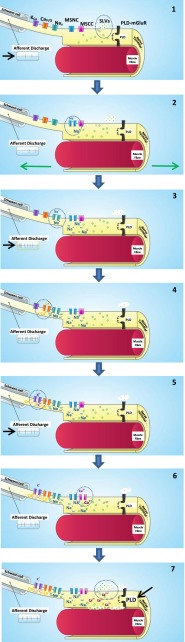
Schematic summarising our current knowledge of the steps (*1–7*) from rest from mechanotransduction, through action potential encoding and firing rate determination, to autogenic sensitivity modulation. Areas of interest in each step are encircled or indicated by *arrows.1*, The myelinated primary afferent axon arrives from the *left*, produces a specialised encoding site at the unmyelinated heminode, then expands to form the sensory terminal proper, enclosing the intrafusal muscle fibre. The afferent discharge rate is shown in the *panel bottom left* (*arrow*). The terminal is the primary site of mechanotransduction through at least one type of mechanosensory channel (*MS*) passing Na^+^ and Ca^2+^. For convenience, these are shown separately (*MSNC* mechanosensitive Na^+^ channel, *MSCC* mechanosensitive Ca^2+^ channel). The terminal, as for all primary mechanosensory nerve endings, contains a population of 50-nm diameter clear vesicles—synaptic-like vesicles (*SLVs*, *green circles*—see text for details). At rest, SLVs undergo spontaneous exocytosis of glutamate (*green dots in dotted area*) to activate the phospholipase d-coupled metabotropic glutamate receptor (*PLD-mGluR*), to enable and maintain ending ability to respond to stretch stimuli. *Abbreviations: Ca*_*P/Q*_ P/Q-type voltage-dependent Ca^2+^ channel, *K*_*Ca*_ Ca^2+^-activated potassium channel, *Na*_*v*_ voltage-dependent sodium channel. *2*, Muscle stretch (*green arrows*) gates the MSNC, and Na^+^ influx depolarises the terminal. *3*, The depolarisation spreads electrotonically to the much narrower heminode encoding region, increasing action potential (*AP*) firing (*black arrow*). Only the voltage-dependent Na^+^ channel component of the AP is shown for simplicity. *4*, The APs trigger the opening of P/Q-type Ca^2+^ channels. *5*, The resulting Ca^2+^ influx opens Ca^2+^-activated K^+^ channels (*K*_*Ca*_), repolarising the heminode region. This negative feedback step moderates the firing rate (*black arrow*). *6*, Simultaneously, the initial stretch also gates a mechanosensitive Ca^2+^ current (through the MSNC or another mechanosensory channel (*MSCC*)), allowing Ca^2+^ influx. *7*, The increased intracellular Ca^2+^ enhances SLV exocytosis of glutamate, further activating the PLD-mGluRs. The resulting increase in PLD activity (*black arrow*) is part of a positive feedback loop (*curved arrows*) that maintains the ability of the ending to respond to subsequent stretches, perhaps by enhancing/maintaining MS channel insertion, through a mechanism that awaits identification. An animated version of this sequence is available online (see Supplementary material, S1)

In summary, spindle responsiveness is both maintained and increased by a positive gain control system whereby SLVs release glutamate in a Ca^2+^- and mechanical activity-dependent manner, activating a PLD-mGluR receptor with a unique pharmacology (Fig. 9; Supplementary material S1). This system controls firing from an approximate doubling of firing rates at one extreme, to abolishing spindle output altogether at the other. Thus, this SLV-glutamate-PLD-mGluR system, operating over a minute-to-hour timescale, seems essential for ensuring spindle functionality in the long term. Given the ubiquitous presence of SLVs in primary mechanosensory nerve terminals, and the very similar glutamate pharmacology we have found in the only two other mechanosensory systems we have examined—lanceolate terminals of the palisade endings of rodent hair follicles [10], and aortic baroreceptors [57]—this gain control system seems likely to be a common feature of all such endings. The recent report of vGluTs in other low-threshold mechanosensory terminals and accessory cells [81, 82] supports this view.

**Fig. 10:**
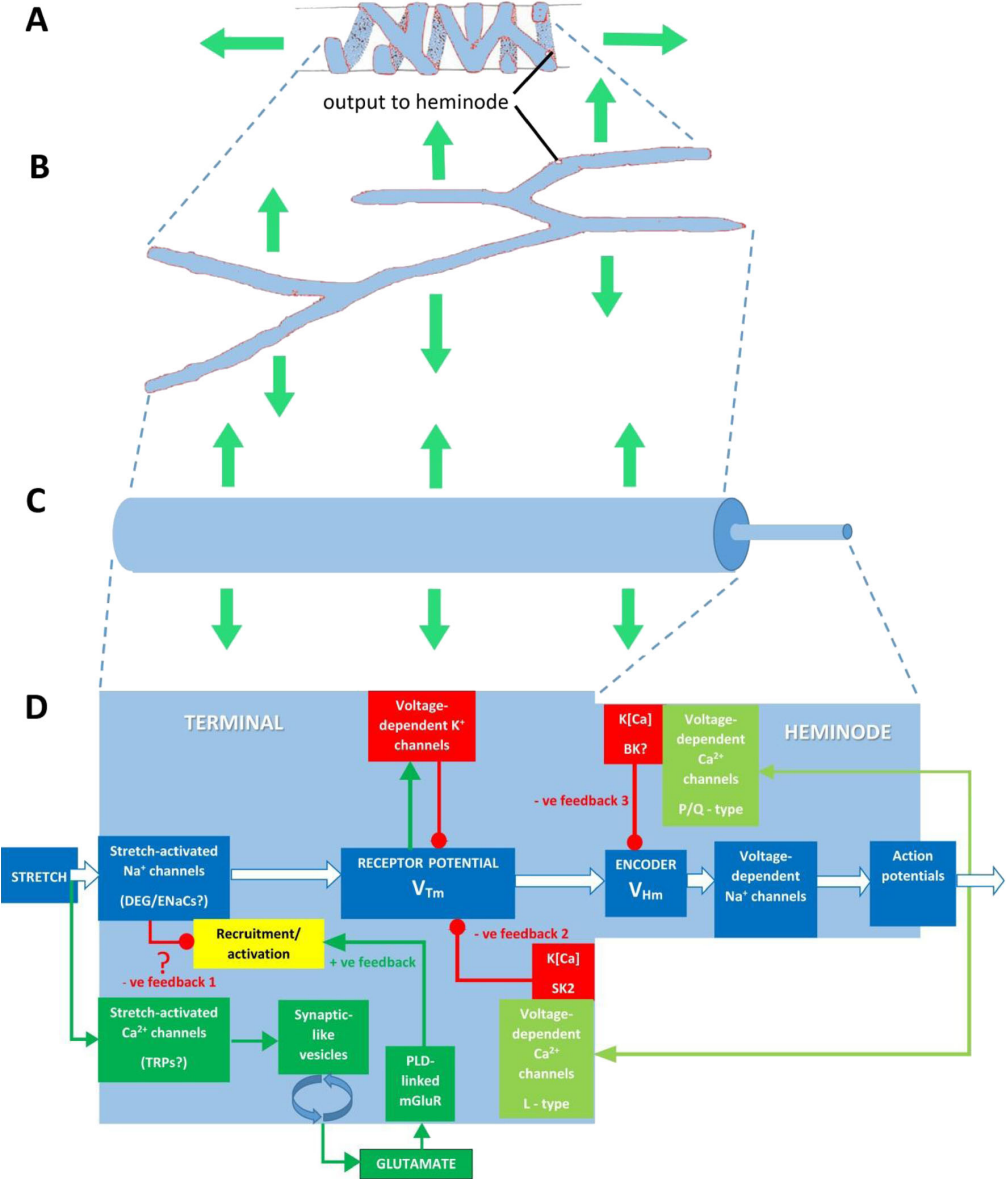
**a**–**d** Progressive geometrical abstraction of a single terminal of a spindle primary ending, leading to a flow-chart summarising the events of mechanosensory transduction. *Green block arrows* in (**a**–**c**) indicate the direction and distribution of stretch applied to the terminal when the primary ending is lengthened during muscle stretch or fusimotor stimulation. **a** A single terminal in its annulospiral form, taken from a primary ending reconstructed from serial sections [8]. Several such terminals typically enclose a single intrafusal muscle fibre. The terminal is connected to its associated heminode by a short, unmyelinated preterminal axonal branch at the point shown. **b** The terminal unrolled and turned through 90°. Note that individual terminals may be repeatedly branched and that the direction of stress during stretch is orthogonal to the long axis of the terminal. **c** A terminal and its associated unmyelinated preterminal branch shown in abstract cylindrical form to indicate the relative diameters of these structures. The smaller preterminal branch to the right is about 1 μm diameter. The lengths, especially that of the much larger terminal to the *left*, are highly variable. **d** Flow chart to illustrate the main events of mechanosensory transduction, as described in this review. The principal feed-forward pathway from stimulus (stretch) to output (action potentials) is shown by the *white block arrows*. We envisage that the overall gain of this pathway is controlled by several feedback pathways: *negative feedback 1* is at present hypothetical and is included to account for the reversible silencing of the primary ending by PCCG-13 inhibition of the PLD-linked mGluR; the *positive feedback* pathway is the well-established SLV/glutamatergic loop; *negative feedbacks 2* and *3* involve different kinds of K[Ca], one located in the terminal, the other in the heminode and both perhaps triggered by action potentials opening voltage-gated Ca channels. *Green lines* and *arrowheads* indicate enhancing/excitatory actions; *red lines* and *circles* indicate reducing/inhibitory actions

Of course, a positive feedback gain control, operating in isolation, would make spindle outputs very unstable, particularly during times of intensive activity. A negative feedback control must also be present to overcome this tendency (Fig. 10). This seems to involve a combination of Ca^2+^ and K[Ca] channels [47, 55, 79], some of which may contribute to the receptor potential itself [40] (Shenton et al., unpublished data), as described in a previous section. Normal activity would activate the voltage-gated Ca^2+^ channels, thereby opening the K^+^ channels and reducing firing. Finally, these complex control systems seem likely to be confined to different loci as protein complexes and also tethered to cytoskeletal elements. We are now exploring one such binding protein, the PDZ-scaffold protein Whirlin. We have recently shown a mutation in Whirlin, which is responsible for the deaf/blindness of Usher’s syndrome, selectively impairs stretch-evoked responsiveness in muscle spindles [23].

## Conclusion

Overall, it is clear that even in the absence of fusimotor activity, spindle stretch-evoked output is the product of complex and sophisticated regulatory gain controls, both positive and negative in nature. Coupled with the polyionic receptor currents and the potential for heteromeric transduction channels, the muscle spindle looks set to continue to keep its reputation as the most complex sensory organ after the special senses, at least for the foreseeable future.

## Electronic supplementary material

Below is the link to the electronic supplementary material.

Supplementary material S1 Animation summarising our current knowledge of the steps from rest, through mechanotransduction, action potential encoding and firing rate determination, to autogenic sensitivity modulation. The myelinated primary afferent axon arrives from the *left*, produces a specialised encoding site at the unmyelinated heminode, then expands to form the sensory terminal proper, enclosing an intrafusal muscle fibre. The afferent discharge rate is shown in the panel (*bottom left*). The terminal is the primary site of mechanotransduction through at least one type of mechanosensory (*MS*) channel, allowing Na^+^ and Ca^2+^ ingress. For convenience, the channel(s) are shown separately (MSNC and MSCC, respectively). The terminal, as for all primary mechanosensory nerve endings, contains a population of 50-nm diameter clear vesicles; i.e. synaptic-like vesicles (SLVs—see main text for details). At rest, SLVs undergo spontaneous exocytosis of glutamate to activate the phospholipase D-coupled metabotropic glutamate receptor (*PLD-mGluR*), to enable and maintain ending ability to respond to stretch stimuli. Muscle stretch gates the MSNC, and Na^+^ influx depolarises the terminal. The depolarisation spreads electrotonically to the much narrower heminode encoding region, increasing action potential firing. Only the voltage-dependent Na^+^ channel component of the AP is shown for simplicity. The APs trigger the opening of P/Q-type Ca^2+^ channels. The resulting Ca^2+^ influx opens Ca^2+^-activated K^+^ channels, repolarising the heminode region. This negative feedback step moderates the firing rate. Simultaneously, the initial stretch also gates a melar Ca^2+^ enhances SLV exocytosis of glutamate, further activating the PLD-mGluRs. The resulting increase in PLD activity is part of a positive feedback loop (*curved arrows*) that maintains the ability of the ending to respond to subsequent stretches, perhaps by enhancing/maintaining MS channel insertion, through a mechanism that awaits identification (Animation designed by IT Media Services, University of Aberdeen) (MP4 16375 kb)
